# Supplementary material for: MetaRibo-Seq measures translation in microbiomes
Source: Nat Commun. 2020 Jun 29;11:3268. doi: 10.1038/s41467-020-17081-z (PMC7324362; doi:10.1038/s41467-020-17081-z)
Supplement: Supplementary file 10 — Supplementary Data 7 [file 41467_2020_17081_MOESM10_ESM.zip › File2/Confidence_VeryHigh_Taxonomy/30534_out.krona.html]

Javascript must be enabled to view this page.

members
magnitude
magnitudeUnassigned
count
unassigned
taxon
rank

30534\_out

91

2
superkingdom
91

phylum
91
1239

186801
class
91

186802
91
order

91
family
541000

18
genus
91

SRS015579\_contig\_number\_15684SRS016018\_contig\_number\_contig-100\_11106.70288SRS017103\_contig\_number\_35581SRS024009\_contig\_number\_contig-100\_3118.81553SRS024331\_contig\_number\_27134SRS049712\_contig\_number\_contig-100\_3245.127658SRS063190\_contig\_number\_contig-100\_2451.92477SRS075821\_contig\_number\_contig-100\_17107.67325SRS077641\_contig\_number\_contig-100\_1582.137993SRS097958\_contig\_number\_contig-100\_3793.139034SRS098061\_contig\_number\_12818SRS1041038\_contig\_number\_contig-100\_2063.97498SRS142923\_contig\_number\_contig-100\_5100.131857SRS143466\_contig\_number\_contig-100\_25471.78139SRS143876\_contig\_number\_contig-100\_2180.234328SRS148874\_contig\_number\_contig-100\_8580.75212SRS149075\_contig\_number\_contig-100\_423.124920SRS893172\_contig\_number\_contig-100\_1688.85796
1263

2293169
species
2

SRS076804\_contig\_number\_20225SRS148319\_contig\_number\_13322

5
species
2293177

SRS020328\_contig\_number\_37019SRS055017\_contig\_number\_contig-100\_14023.150044SRS097889\_contig\_number\_1511SRS143780\_contig\_number\_786SRS149181\_contig\_number\_27856

2
species
2293216

SRS1041139\_contig\_number\_contig-100\_2550.41649SRS104311\_contig\_number\_19656


SRS013687\_contig\_number\_15558SRS046717\_contig\_number\_contig-100\_1915.87760SRS893270\_contig\_number\_contig-100\_1186.135619
41978
species
3

2293165
species
4

SRS016203\_contig\_number\_contig-100\_1732.1732SRS075878\_contig\_number\_20120SRS104400\_contig\_number\_contig-100\_422.346020SRS143372\_contig\_number\_1406

6
species
2293161

SRS011405\_contig\_number\_889SRS045826\_contig\_number\_14064SRS098571\_contig\_number\_26299SRS1055049\_contig\_number\_3223SRS143523\_contig\_number\_9559SRS893230\_contig\_number\_contig-100\_1891.78301


SRS011239\_contig\_number\_23251SRS015065\_contig\_number\_21294SRS015663\_contig\_number\_contig-100\_3541.204106SRS016629\_contig\_number\_contig-100\_270.38023SRS016989\_contig\_number\_405SRS019286\_contig\_number\_contig-100\_1413.50546SRS023914\_contig\_number\_contig-100\_536.160882SRS042628\_contig\_number\_10783SRS051031\_contig\_number\_14180SRS052027\_contig\_number\_12737SRS054905\_contig\_number\_contig-100\_1127.154865SRS062427\_contig\_number\_5382SRS064276\_contig\_number\_38948SRS1041036\_contig\_number\_contig-100\_2241.2242SRS1041091\_contig\_number\_8895SRS104636\_contig\_number\_contig-100\_2169.164095SRS1055069\_contig\_number\_contig-100\_23103.60294SRS147139\_contig\_number\_3088SRS148424\_contig\_number\_contig-100\_1239.197106SRS893358\_contig\_number\_contig-100\_3127.80074SRS893378\_contig\_number\_contig-100\_1910.123288SRS971275\_contig\_number\_29539SRS971276\_contig\_number\_contig-100\_805.218261
2293157
species
23

2293178
1
species

SRS144506\_contig\_number\_31557


SRS012273\_contig\_number\_34376SRS016495\_contig\_number\_2431SRS018541\_contig\_number\_contig-100\_236.94496SRS020394\_contig\_number\_17668SRS022071\_contig\_number\_36582SRS053214\_contig\_number\_19546SRS077294\_contig\_number\_17935SRS144297\_contig\_number\_contig-100\_3875.110746SRS144714\_contig\_number\_26074
2293149
species
9

1
species
165186

SRS893252\_contig\_number\_contig-100\_7226.51338

12
species
2293194

SRS014923\_contig\_number\_43032SRS019178\_contig\_number\_contig-100\_931.43301SRS024492\_contig\_number\_contig-100\_3671.108360SRS045195\_contig\_number\_7202SRS050925\_contig\_number\_contig-100\_3169.186912SRS051882\_contig\_number\_21709SRS063489\_contig\_number\_contig-100\_10623.46277SRS101433\_contig\_number\_contig-100\_15984.56647SRS1041033\_contig\_number\_contig-100\_4447.159985SRS1041133\_contig\_number\_15795SRS142712\_contig\_number\_5158SRS893279\_contig\_number\_4419

1160721
species
3

SRS015854\_contig\_number\_contig-100\_4192.72348SRS147377\_contig\_number\_13063SRS147919\_contig\_number\_21496


SRS893373\_contig\_number\_11712
2293156
1
species


SRS014855\_contig\_number\_15490
species
1
1262962
